# Integrating evidence synthesis into doctoral research: A guide for family medicine and primary care

**DOI:** 10.4102/phcfm.v17i2.5198

**Published:** 2025-12-04

**Authors:** Klaus B. von Pressentin, Jacob S. Shabani, Taryn Young

**Affiliations:** 1 Division of Family Medicine, Department of Family, Community and Emergency Care, Faculty of Health Sciences, University of Cape Town, Cape Town, South Africa; 2 Department of Family Medicine, Medical College, East Africa, Aga Khan University, Nairobi, Kenya; 3 Division of Epidemiology and Biostatistics, Department of Global Health, Faculty of Medicine and Health Sciences, Stellenbosch University, Tygerberg, South Africa

**Keywords:** evidence synthesis, primary care, doctoral education, research design, knowledge translation, scoping review

## Abstract

Given the increased complexity of healthcare needs, evidence-informed practices are needed, now more than ever. Combining the best available research evidence, the perspectives of patients and communities, and the voices of healthcare workers in guiding policy and practice is essential. All of us involved in providing and strengthening family medicine and primary care need to be good consumers (users) of research, and some will be good producers (doers) of research. In both using and doing research, a helpful starting point is evidence synthesis – a form of secondary research that collates primary research on the same research question. This short report outlines when and how to incorporate evidence synthesis into doctoral work, highlighting methodological considerations, ethical principles and reporting standards. Practical tips and decision points are provided to support relevance, rigour and impact. Thoughtful integration of evidence synthesis – whether by using existing reviews or conducting new ones – enables doctoral researchers to contribute meaningfully to evidence-informed primary care practice and policy.

## Introduction

Evidence-informed practices are widely adopted to improve health care policy and professional care and services activities, including within the discipline of family medicine and primary care. These practices integrate research evidence with healthcare professional expertise and client perspectives to improve decision-making and health outcomes. Key to considering research as part of this process is to ensure that it represents the totality of research on the topic and is up to date, robust and relevant to the setting. Evidence synthesis can be defined as the review of what is known from existing research using systematic and explicit methods to clarify the evidence base.^1^ In other words, evidence synthesis clarifies what is known and not known about a particular research question.

Evidence synthesis does not exist in isolation; it forms part of a broader evidence ecosystem encompassing evidence generation, synthesis and translation.^2^ This ecosystem reflects the dynamic interplay between producing new knowledge, consolidating existing findings and translating evidence into policy and practice.^3^ Research translation seeks to bridge the gap between research and decision-making involving diverse actors and activities that extend beyond traditional disciplinary boundaries.^4^ Understanding this ecosystem is critical for doctoral researchers, as it situates their work within the continuum of knowledge production and use.

The doctoral journey offers valuable opportunities to both build upon existing research and conduct new research. This facilitates clarification of knowledge gaps and positions their work within the broader scientific landscape. As the volume of published literature continues to expand, it also becomes increasingly important to reduce avoidable waste in research, conducting timely, relevant, rigorous research with clearly unbiased reporting.^5,6^ Doctoral researchers can play a key role in reducing research waste. For example, this could include incorporating an existing rigorously conducted synthesis to inform the different phases of their doctoral research, such as in mapping and identifying key research gaps on a topic, determining appropriate outcome measures and describing existing methodological approaches, among others. Unfortunately, among these existing evidence syntheses, there is also an increasing number of irrelevant and redundant reviews, which makes quality appraisal even more vital.^7^

Evidence synthesis can therefore be incorporated into a doctoral research project in two complementary ways: by using existing reviews to inform the research process and by conducting a new synthesis as part of the thesis output. The latter approach, however, should be undertaken judiciously. The first step is to assess whether relevant syntheses already exist and determine if a new synthesis is warranted.^5,8^ This decision requires a nuanced understanding of the various synthesis methodologies, their respective strengths and limitations and the specific research needs they address.

This short report provides practical guidance for making these decisions and aims to equip doctoral researchers with approaches to locate, critically appraise, interpret and integrate existing evidence. We will also provide a brief outline on how to conduct a scoping review as the most frequently encountered form of evidence synthesis in doctoral research.

## Using existing evidence synthesis

Sir Ian Chalmers stated that:

The results of a particular research study cannot be interpreted with any confidence unless they have been considered together with the results of other studies addressing the same or similar questions.^9^

**FIGURE 1:**
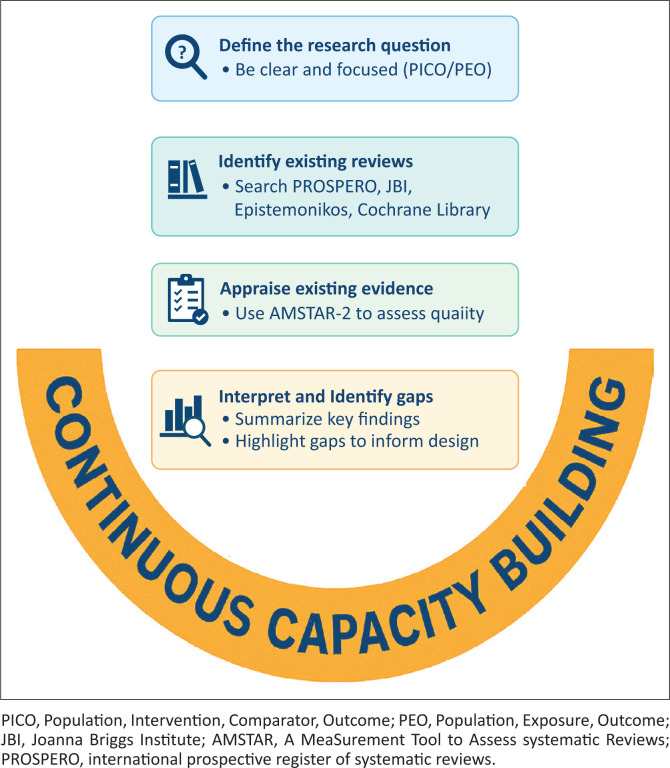
Steps for appraising and interpreting existing evidence.

All researchers thus need a structured process of taking stock of existing research – both primary and secondary research – to have not only a summary of previous findings but also a critical examination and synthesis of existing reports – giving the current state of knowledge with the strengths and limitations of the underlying research.^10^ Evidence synthesis, also known as secondary research, uses methods to identify, collate, appraise and interpret existing research. When developing your research rationale, consider starting with existing evidence synthesis to inform your engagement with the existing literature. In this section, we will provide you with helpful tips aligned with the steps depicted in Figure 1.

### Clarify your research question

Begin by framing a clear, focused question using structured frameworks. A well-defined question ensures efficient searching and appraisal. Utilise frameworks such as PICO (Population, Intervention, Comparator, Outcome) or PEO (Population, Exposure, Outcome) to structure your question. For example, ‘In adults with hypertension (Population), does telehealth monitoring (Intervention) compared to usual care (Comparator) improve blood pressure control (Outcome)?’

### Identify existing evidence synthesis

Depending on the methodological approach, there are different types of evidence synthesis. You will need to decide which types you are trying to identify. Specialised databases and registries for consideration are highlighted below:

- PROSPERO and Open Science Framework – International prospective registers of systematic reviews currently in progress.^11^
- Joanna Briggs Institute (JBI) Evidence Synthesis – Methodologically diverse reviews, including qualitative and mixed methods.^12^
- Epistemonikos – Cochrane and non-Cochrane systematic reviews and overviews of reviews of health care research.^13^
- Cochrane Library – High-quality systematic reviews of healthcare interventions and diagnostic tests.^14^

In Table 1, we include some popular review types and their key features and provide a few examples to illustrate these differences. Designing a search strategy can be a complex undertaking, and doctoral researchers may benefit from the skills and expertise of a medical or health science librarian.

**TABLE 1:** Comparison of evidence synthesis types.

Type of evidence synthesis	Key characteristics and distinguishing features	What is a good example?
Scoping review	Explores the extent, range and nature of research activity; identifies gaps in the evidence base; often used to clarify key concepts or definitions.	Models of integrated care for multi-morbidity assessed in systematic reviews: a scoping review.^15^
Systematic reviews	Synthesises and appraises all the evidence to address a single well-defined question and sometimes performs a metanalysis of the combined data.	Community-based active case-finding interventions for tuberculosis: a systematic review.^16^
Rapid review	Uses streamlined review methods; may limit search databases, timeframes or appraisal depth to produce faster results. The key is to do it quickly.	Transmission of respiratory viruses when using public ground transport: a rapid review to inform public health recommendations during the COVID-19 pandemic.^17^
Overview review	Synthesises and compares findings across systematic reviews, with a narrower, more methodologically rigorous focus on synthesising reviews on a single topic.	What are the effects of teaching EBHC at different levels of health professions education? An updated overview of systematic reviews.^18^
Umbrella review	It is a broader, more integrative review, combining different types of reviews (and sometimes primary studies) to give a holistic summary of a field.	Primary health care quality indicators: An umbrella review.^19^

Note: Please see the full reference list of the article Von Pressentin KB, Shabani JS, Young T. Integrating evidence synthesis into doctoral research: A guide for family medicine and primary care. Afr J Prm Health Care Fam Med. 2025;17(2), a5198. https://doi.org/10.4102/phcfm.v17i2.5198, for more information.

COVID-19, coronavirus disease 2019; EBHC, Evidence-Based Health Care.

### Appraise

When reading an evidence synthesis, one assesses the rigour of the methods used first before looking at the results. Validated tools, such as AMSTAR-2, a 16-item critical appraisal tool, are used to evaluate the methodological quality of existing syntheses that include randomised or non-randomised studies of healthcare interventions.^20^ Key domains to check include protocol registration, comprehensive search strategy, risk of bias assessment and transparency in reporting.

### Interpret and identify gaps for new research

Then, read, interpret and make sense of the findings. Critically analyse the findings to identify gaps; consider the:

- **Scope**: Are certain populations, interventions or outcomes underrepresented?
- **Currency**: Is the review outdated (more than 3–5 years old)?
- **Limitations**: Was grey literature or non-English studies excluded?

Evaluating these gaps may inform the design of your doctoral study, such as justifying updating an existing review or designing a new synthesis or primary study to address the identified gaps.

Continuous capacity building on how to find and appraise existing research underpins these steps, as depicted in Figure 1. It is essential to enable individuals and institutions to have the software, knowledge and skills to identify and consider the application of evidence syntheses.^21^ It involves strengthening research and analytical skills while promoting stakeholder engagement and communication.

## Conducting a new evidence synthesis

Beyond using existing evidence synthesis, one can conduct a new synthesis if it is identified as needed. Incorporating a scoping or systematic review into a doctoral project is common, as it enhances the process for taking stock of existing research to inform the doctoral research direction. At many institutions, such reviews may be published as standalone outputs, often as one of the articles or a chapter in the thesis.

Carrying out a high-quality evidence synthesis requires careful planning, adherence to established guidelines and a clear understanding of methodological steps. Although the results of an evidence synthesis can be vital to a doctoral project, many underestimate the effort involved. Systematic reviews, in particular, are time-intensive and methodologically demanding, requiring specialised skills in literature searching, screening and appraisal. The support of an information specialist or librarian can be invaluable for developing search strategies and managing data efficiently.

In this section, we will focus on guidance for doing a scoping review. Other formats, such as systematic or rapid reviews, may also be appropriate depending on the research question.^22^ We recommend that doctoral researchers consult their supervisors to select the most suitable synthesis method. The decision to conduct a scoping review should be made carefully, considering both the research question and the resources available to them.

Scoping reviews are based on interpretivist and constructivist paradigms, which reject the idea of absolute truth and focus on generating knowledge through interactions among researchers, data and context.^23^ Their iterative and adaptable process allows for refining inclusion criteria during screening and analysis, fitting well with qualitative traditions. Researchers should consider the epistemological principles underlying scoping reviews and ensure they align with their worldview. Typically, scoping reviews encompass a wide range of literature, including grey literature, to comprehensively map evidence. This approach often yields nuanced findings, necessitating a team effort in data analysis to identify patterns and differences. Ultimately, because of their subjective nature, scoping reviews require transparent and defensible decision-making, with researchers critically reflecting on how their perspectives influence interpretation and methodological choices.

## Resources for a scoping review

Next follows a short discussion on the various resources that are needed for conducting a scoping review.

### Team

A scoping review needs a research team with the right expertise and tools. Librarians are essential members of scoping review teams because their expertise helps develop a thorough search strategy that finds key articles and other relevant resources, setting the foundation for the next steps. A content expert also adds value by helping the team understand the scope and depth of the topic, choosing relevant search terms and keywords, and creating the data extraction form. A team member with expertise in scoping review methods can help determine if a scoping review is suitable for the research question and guide the team through each stage. This might include decisions about team membership and methods to ensure rigour, such as developing the data extraction form. Content experts may also assist in interpreting results after data analysis or suggest alternative approaches. At least two reviewers are needed to assess titles, abstracts and full articles independently; in cases of disagreement, a third reviewer may be involved. If having two reviewers is impractical, one can do the review while a second (possibly a supervisor of the doctoral study) verifies some of the articles. Having sufficient human resources is crucial for completing a scoping review efficiently, as delays can render the search results outdated. If that occurs, the search must be rerun to include articles published after the initial search period.

### Time

Conducting a scoping review requires significant time, as reviewers must carefully read each title and abstract to determine eligibility based on inclusion criteria. Simultaneously, the team needs time to discuss which aspects of the included studies to extract and to develop a data extraction form aligned with those aspects. For included articles, reviewers must read the full text to identify relevant excerpts for extraction. Additionally, both the study selection and data extraction phases involve calibration exercises – meetings where reviewers clarify inclusion criteria, resolve discrepancies and aim for at least 90% agreement.

### Tools

Tools assist in scoping review phases, such as organising references, identifying duplicates and documenting team justifications for inclusion or exclusion. Rayyan, a free mobile and web app, enables study selection among reviewers, tracking decisions with space for brief justifications, while allowing blinded collaboration. Covidence, similar to Rayyan, supports scoping reviews. See also the details in the approach steps described below.

## Approach

Arksey and O’Malley’s key articles on scoping reviews proposed a five-stage framework, later extended to six stages, to guide the conduct of a scoping review.^24^ Other methods or frameworks, such as JBI and Levac et al., should also be considered, and it is essential to state the choice in the methods section of the doctoral study protocol.^25,26^ This type of review focuses on mapping existing literature and identifying gaps rather than performing a more focused systematic review. Develop a protocol in advance, detailing objectives, eligibility criteria, search strategy, screening methods and plans for synthesis.^27^

### Stage 1: Identify the research question

Begin by defining your review’s aim and scope. Clarify if you aim to explore evidence, map uncertainties or identify gaps. Clearly stated objectives keep subsequent steps focused and aligned with the study’s aim and epistemology. Researchers should formulate a broad question with a clear scope, defining the concept, population and health outcomes to clarify focus and develop an effective search strategy. Use the P (population), C (concept), C (context) (PCC) framework to structure the question. In a scoping review, the main question may be divided into several sub-questions aligned with the objectives.

### Stage 2: Identify relevant studies

A strength of scoping studies is their breadth of evidence and ability to consider the extent of evidence in a particular field. However, practical issues like time, funding and resources often force researchers to balance feasibility, breadth and comprehensiveness. Ideally, researchers should ensure that feasibility does not hinder their ability to answer the research question or meet the study’s purpose. Additionally, assembling a team with methodological and contextual expertise is advised for decisions on scope, and justifications should be provided when scope is limited, acknowledging potential study limitations. Designing a comprehensive search strategy – including key databases and grey literature – with an information specialist can improve search efficiency. Define the eligibility criteria (or inclusion criteria) in alignment with the review’s objective and question(s) by using the PCC framework.

### Stage 3: Select the studies

Apply inclusion and exclusion criteria uniformly across all records. Utilise tools such as Rayyan for blinded screening or Covidence for workflow management, thereby supporting dual screening to minimise bias.^28^ It is advisable that multidisciplinary teams conduct transparent, reproducible scoping studies. Teams should initially meet to discuss study inclusion and exclusion criteria, refine search strategies based on abstracts and review full articles. At least two researchers should independently screen abstracts, meeting at stages to address issues and refine strategies. Two reviewers should also independently examine full articles, consulting a third reviewer if disagreements occur.

### Stage 4: Chart the data

Develop and pilot a standardised charting form before full data extraction. The research team creates a data-charting form or data extraction tool to identify which variables to extract for answering the research question. Charting can be seen as an iterative process. Uncertainty about the data to extract can be managed by starting the charting process and then refining the form after familiarisation. It is recommended that two researchers independently extract data from the first 5 to 10 studies using the form, then meet to check for consistency with the research question and purpose. Use reference managers such as Zotero or EndNote and data management tools such as Excel or Covidence to organise extracted information efficiently.^28,29^

### Stage 5: Collate, summarise and report the results

Plan your synthesis approach a priori. Unlike a systematic review, a scoping study aims to provide an overview of all reviewed materials and focuses on presentation, not synthesising evidence or aggregating findings. It may use a framework to narrate the literature, but it does not evaluate the quality of the evidence. Scoping reviews often use descriptive or thematic mapping. Transparency in reporting is a cornerstone of ethical evidence synthesis. Doctoral researchers should adhere to established reporting guidelines such as Preferred Reporting Items for Systematic Reviews and Meta-Analyses for scoping reviews (PRISMA-ScR) for scoping reviews, as well as resources from the EQUATOR Network.^30,31,32^ The PRISMA flow diagram is an example of required reporting, as it shows the process of selecting the articles for the synthesis. Ethical reporting also involves acknowledging limitations, disclosing conflicts of interest and ensuring findings are disseminated in accessible formats to support evidence-informed decision-making.

### Stage 6: Consultation stage

Incorporating input from key stakeholders, such as practitioners and policymakers, enhances evidence syntheses by integrating stakeholder consultation as a key component of knowledge translation in scoping reviews. Researchers should define the consultation’s purpose, such as sharing findings, validation, or guiding future research. Use stage five results as a base, allowing stakeholders to add context and apply their expertise. Specify stakeholder types, data collection methods (like focus groups, interviews and surveys), analysis plans and consensus-building approaches (Delphi method and nominal group technique).^33^ Because consultation involves orienting stakeholders on the study’s purpose, findings and dissemination, it also acts as a knowledge transfer tool, addressing concerns about practical use and translating knowledge. This stage aids in developing dissemination strategies, boosting the study’s value.

### Ethical considerations

Although scoping reviews typically do not require formal ethics approval, ethical conduct remains a critical component of scholarly integrity. Some institutions may require students to apply for a waiver from the Health Research Ethics Committee or Institutional Review Board to include a scoping review in their thesis. Ethical practice begins with avoiding redundancy and research waste by conducting preliminary searches in registries such as PROSPERO, JBI, Epistemonikos and the Cochrane Library. Researchers should also consider the review’s relevance and potential contribution to the evidence ecosystem, asking: Who is the intended audience – clinicians, educators, policymakers or researchers – and how will the findings be used? Registering your protocol on platforms such as Open Science Framework or PROSPERO enhances transparency and reduces duplication. Protocol development and registration are considered best practice for both systematic and scoping reviews.^27^

## Training and resources

Developing competencies for ethical and methodologically sound reviews requires structured training and mentorship. While postgraduate training in family medicine in the sub-Saharan African region includes mastery of evidence-based practice (EBP), these programmes focus mainly on the foundational principles of evidence-based medicine linked to clinical practice and improving health services. Evidence-based practice or evidence-informed care merges patients’ values and preferences with clinical expertise and the best available evidence to provide the highest quality care possible to patients. A recent scoping review highlighted five strategies for research capacity building by family physicians: training and mentoring, networking, developing strategic models, strategic communication and knowledge transfer.^34^ These can enhance research engagement and better integrate research into clinical practice. Implementing these strategies fosters a stronger research culture in primary care, improving evidence-based care.

However, doctoral researchers require more advanced skills and would benefit from additional training in methods for conducting robust and trustworthy evidence synthesis.^7^ This approach involves engaging with experienced researchers in the evidence community, participating in peer collaborations and utilising online resources linked to global and regional evidence synthesis institutions and networks.^35^ Doctoral researchers are also encouraged to engage with discipline-specific networks, including the Primary Care and Family Medicine (PRIMAFAMED) network, World Family Doctor Organisation (WONCA) and the North American Primary Care Research Group (NAPCRG), as well as link with other related disciplines, networks and organisations involved in building capacity at individual and institutional levels in using and conducting evidence synthesis.^36,37,38^

## Conclusion

Engaging with existing evidence syntheses supports doctoral researchers in developing their projects. It helps prevent duplication by identifying topics that have already been studied – an important consideration given that meta-research consistently shows researchers often repeat work when sufficient evidence already exists. Evidence synthesis also clarifies research questions and highlights knowledge gaps before new studies are undertaken. By systematically gathering, analysing and summarising existing evidence, doctoral researchers can better understand the current state of knowledge and refine their research focus accordingly. When well-conducted and up to date, such syntheses provide a solid foundation for designing new studies; when reviews are rigorous but outdated, they signal opportunities for review updates. Beyond shaping individual projects, integrating scoping or systematic reviews into doctoral work contributes to a culture of transparency, collaboration and EBP. By selecting appropriate review methods, adhering to high methodological standards and sharing findings accessibly with clinicians, educators and policymakers, doctoral researchers can ensure their work builds meaningfully on prior knowledge and advances the discipline as a whole.
